# *catena*-Poly[[(5,5,7,12,12,14-hexa­methyl-1,4,8,11-tetra­aza­cyclo­tetra­deca-1,7-diene)copper(II)]-μ-chlorido-[dichloro­cuprate(II)]-μ-chlorido]

**DOI:** 10.1107/S1600536812024932

**Published:** 2012-06-13

**Authors:** Bohari M. Yamin, Wafiuddin Ismail, Jean-Claude Daran

**Affiliations:** a Low Carbon Economy Research Group, School of Chemical Sciences and Food Technology, Universiti Kebangsaan Malaysia, UKM 43600 Bangi Selangor, Malaysia; b School of Chemical Sciences and Food Technology, Universiti Kebangsaan Malaysia, UKM 43600 Bangi Selangor, Malaysia; c Laboratoire de Chimie de Coordination, UPR5241, 205 Route de Narbonne 31077, Toulouse Cedex 04, France

## Abstract

In the title compound, [Cu_2_Cl_4_(C_16_H_32_N_4_)]_*n*_, the central Cu^II^ anion of the macrocyclic complex cation is weakly linked to two Cl atoms of the tetrachloridocuprate anion with Cu—Cl distances of 3.008 (3) and 3.220 (3) Å, respectively, forming a chain parallel to [10-1]. The geometry of the Cu–macrocyclic complex is distorted octa­hedral with the bridging Cl atoms occupying the axial position at an angle of 173.44 (7)° about the central Cu^II^ atom. The tetrachloridocuprate anion adopts a distorted tetra­hedral geometry. In the crystal, the chain is stabilized by intra- and inter­molecular N—H⋯Cl hydrogen bonds.

## Related literature

For related crystal structures, see: Shi & He (2011 ▶); Lu *et al.* (1981 ▶); Podberezskaya *et al.* (1986 ▶). For the preparation, see: Curtis & Hay (1966 ▶); Curtis *et al.* (1975 ▶). For bond-length and angle data, see: Allen *et al.* (2003 ▶); Orpen *et al.* (1989 ▶).
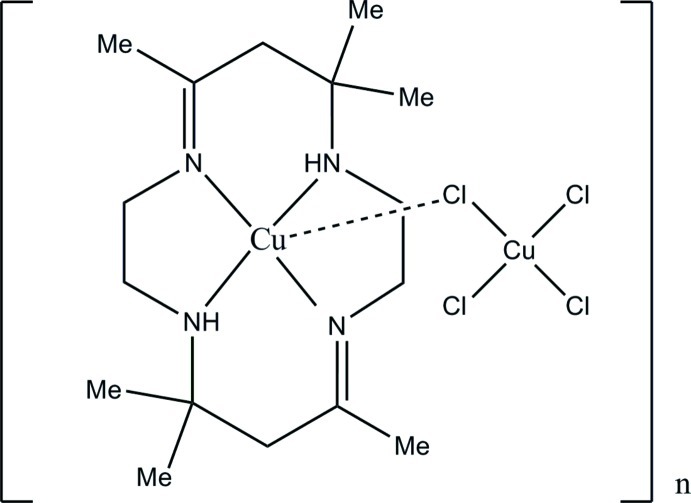


## Experimental

### 

#### Crystal data

[Cu_2_Cl_4_(C_16_H_32_N_4_)]
*M* *_r_* = 549.34
Monoclinic, 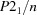

*a* = 9.660 (3) Å
*b* = 15.039 (4) Å
*c* = 16.160 (5) Å
β = 102.424 (7)°
*V* = 2292.6 (12) Å^3^
*Z* = 4
Mo *K*α radiation
μ = 2.33 mm^−1^
*T* = 298 K
0.50 × 0.49 × 0.19 mm

#### Data collection

Bruker SMART APEX CCD area-detector diffractometer
Absorption correction: multi-scan (*SADABS*; Bruker, 2000 ▶) *T*_min_ = 0.389, *T*_max_ = 0.666
11336 measured reflections
3956 independent reflections
2763 reflections with *I* > 2σ(*I*)
*R*_int_ = 0.051

#### Refinement

*R*[*F* ^2^ > 2σ(*F* ^2^)] = 0.056
*wR*(*F* ^2^) = 0.188
*S* = 1.06
3952 reflections
241 parameters
H-atom parameters constrained
Δρ_max_ = 0.93 e Å^−3^
Δρ_min_ = −0.80 e Å^−3^

### 

Data collection: *SMART* (Bruker, 2000 ▶); cell refinement: *SAINT* (Bruker, 2000 ▶); data reduction: *SAINT*; program(s) used to solve structure: *SHELXTL* (Sheldrick, 2008 ▶); program(s) used to refine structure: *SHELXTL*; molecular graphics: *SHELXTL*, *ORTEPIII* (Burnett & Johnson, 1996 ▶) and *ORTEP-3 for Windows* (Farrugia, 1997 ▶); software used to prepare material for publication: *SHELXTL*, *PARST* (Nardelli, 1995 ▶) and *PLATON* (Spek, 2009 ▶).

## Supplementary Material

Crystal structure: contains datablock(s) global, I. DOI: 10.1107/S1600536812024932/bq2363sup1.cif

Structure factors: contains datablock(s) I. DOI: 10.1107/S1600536812024932/bq2363Isup2.hkl

Additional supplementary materials:  crystallographic information; 3D view; checkCIF report

## Figures and Tables

**Table 1:** Hydrogen-bond geometry (Å, °)

*D*—H⋯*A*	*D*—H	H⋯*A*	*D*⋯*A*	*D*—H⋯*A*
N1—H1⋯Cl3^i^	0.91	2.62	3.498 (6)	163
N1—H1⋯Cl4^i^	0.91	2.94	3.527 (5)	123
N3—H3⋯Cl1	0.91	2.62	3.479 (6)	159
N3—H3⋯Cl2	0.91	2.84	3.394 (5)	121

Symmetry code: (i) 
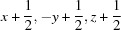
.

**Fig. 1.**
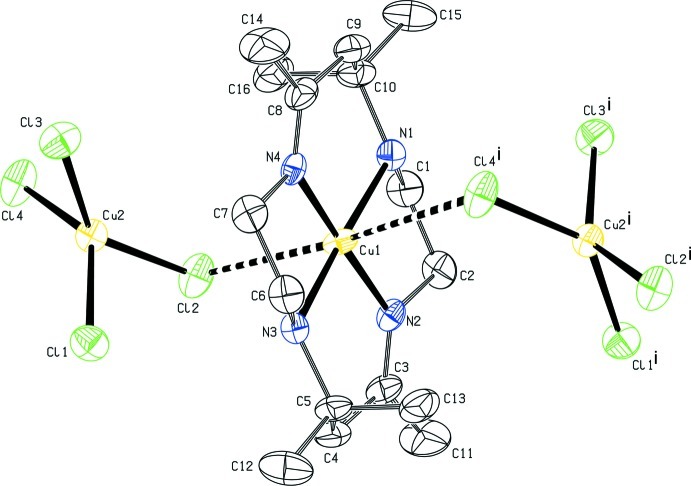
The molecular structure of (I), with the atom labeling scheme. Displacement ellipsoids are drawn at the 30% probability level. H atoms have been omitted for clarity. The long Cu—Cl interactions are shown as dashed lines. (i): 1/2+x,1/2-y,1/2+z.

## supplementary crystallographic information

### Comment

The 14-membered macrocyclic ring 5,7,7,12,14,14,-hexamethyl-1,4,8,11-tetra azacyclotetradeca-4,11-diene (*L*) formed complexes with copper in a variety of coordination modes depending on the copper salts used and other reagents. The salt type complexes such as [CuBr(*L*)]Br.2H_2_O (Shi & He, 2011),[Cu(*L*)] ClO_4_ (Lu *et al.*, 1981) and [CuI(*L*)]IH_2_O (Podberezskaya *et al.*, 1986) are common examples when the ligand was reacted with CuBr_2_, Cu(ClO_4_)_2_, and CuI_2_, respectively. In contrast, a one-dimensional polymeric chain, [Cu(*L*)CuCl_4_]_n_, was obtained when ammonium tetrachlorocuprate(II) was employed to react with the ligand (Fig.1). The Cu1 atom is coordinated to the opposite pair of amino (N1 and N3) and imino (N2 and N4) nitrogen atoms in the same way as in the examples. However, the central Cu1 atom is connected to the Cl2 atom of the tetrachlorocuprate(II) and also to the symmetrically related Cl4^i^ with Cu1—Cl2 and Cu1—Cl4^i^ distances of 3.008 (3) and 3.220 (3) Å, respectively (Fig. 1). As the result, the Cu1 atom formed a distorted octahedral geometry with Cl2 and Cl4^i^ occupy the axial position at an angle about the Cu1 atom of 173.44 (7)°. The bridging angle of Cu1—Cl2—Cu2 and Cu1—Cl4^i^—Cu2^i^ are 105.62 (9)° and 102.43 (8)°, respectively. The tetrachlorocuprate has a distorted tetrahedral geometry with angles about the Cu2 atom between 94.39 (9)° and 139.23 (10)°. The bond lengths and angles are in normal ranges (Allen *et al.*, 2003; Orpen *et al.*, 1989) and comparable to those in the example complexes. In the crystal structure, the molecular chain is also stabilized by intramolecular and intramolecular hydrogen bonds (symmetry codes as in Table 2).

### Experimental

All solvent and chemicals were of analytical grade and were used without purification. The macrocylic compound was prepared according to the literature methods (Curtis & Hay, 1966; Curtis *et al.*, 1975). Equimolar amount of the macrocylic ligand (81 mg) and NH_4_CuCl_4_ (52 mg) was mixed with ethanol and stirred for about 20 minutes. Some single crystals were obtained from the solution after one week of evaporation (yield 61%, m.p 670.3–671.0 K).

### Refinement

H atoms on the parent carbon atoms were positioned geometrically with C—H= 0.96–0.98 Å and constrained to ride on their parent atoms with *U*_iso_(H)= *xU*_eq_(parent atom) where *x*=1.5 for CH_3_ group and 1.2 for CH_2_ and CH groups. The H atom attached to nitrogen were located on difference Fourier but introduced in calculated positions and treated as riding with N—H= 0.91 Å and *U*_iso_(H)= 1.2*U*_eq_(N). A rotating group model was applied to the methyl group.

### Crystal data

**Table:**

[Cu_2_Cl_4_(C_16_H_32_N_4_)]	*F*(000) = 1128
*M**_r_* = 549.34	*D*_x_ = 1.592 Mg m^−^^3^
Monoclinic, *P*2_1_/*n*	Melting point = 671.0–670.3 K
Hall symbol: -P 2yn	Mo *K*α radiation, λ = 0.71073 Å
*a* = 9.660 (3) Å	Cell parameters from 4236 reflections
*b* = 15.039 (4) Å	θ = 1.8–25.0°
*c* = 16.160 (5) Å	µ = 2.33 mm^−^^1^
β = 102.424 (7)°	*T* = 298 K
*V* = 2292.6 (12) Å^3^	Block, violet
*Z* = 4	0.50 × 0.49 × 0.19 mm

### Data collection

**Table:**

Bruker SMART APEX CCD area-detector diffractometer	3956 independent reflections
Radiation source: fine-focus sealed tube	2763 reflections with *I* > 2σ(*I*)
Graphite monochromator	*R*_int_ = 0.051
Detector resolution: 83.66 pixels mm^-1^	θ_max_ = 25.0°, θ_min_ = 1.8°
ω scan	*h* = −11→11
Absorption correction: multi-scan (*SADABS*; Bruker, 2000)	*k* = −17→16
*T*_min_ = 0.389, *T*_max_ = 0.666	*l* = −15→19
11336 measured reflections	

### Refinement

**Table:**

Refinement on *F*^2^	Primary atom site location: structure-invariant direct methods
Least-squares matrix: full	Secondary atom site location: difference Fourier map
*R*[*F*^2^ > 2σ(*F*^2^)] = 0.056	Hydrogen site location: inferred from neighbouring sites
*wR*(*F*^2^) = 0.188	H-atom parameters constrained
*S* = 1.06	*w* = 1/[σ^2^(*F*_o_^2^) + (0.0821*P*)^2^ + 7.8527*P*] where *P* = (*F*_o_^2^ + 2*F*_c_^2^)/3
3952 reflections	(Δ/σ)_max_ < 0.001
241 parameters	Δρ_max_ = 0.93 e Å^−^^3^
0 restraints	Δρ_min_ = −0.80 e Å^−^^3^

### Special details

**Table:**

Geometry. All esds (except the esd in the dihedral angle between two l.s. planes) are estimated using the full covariance matrix. The cell esds are taken into account individually in the estimation of esds in distances, angles and torsion angles; correlations between esds in cell parameters are only used when they are defined by crystal symmetry. An approximate (isotropic) treatment of cell esds is used for estimating esds involving l.s. planes.
Refinement. Refinement of *F*^2^ against ALL reflections. The weighted *R*-factor *wR* and goodness of fit *S* are based on *F*^2^, conventional *R*-factors *R* are based on *F*, with *F* set to zero for negative *F*^2^. The threshold expression of *F*^2^ > σ(*F*^2^) is used only for calculating *R*-factors(gt) *etc*. and is not relevant to the choice of reflections for refinement. *R*-factors based on *F*^2^ are statistically about twice as large as those based on *F*, and *R*- factors based on ALL data will be even larger.

### Fractional atomic coordinates and isotropic or equivalent isotropic displacement parameters (Å^2^)

**Table:**

	*x*	*y*	*z*	*U*_iso_*/*U*_eq_	
C1	0.7323 (8)	0.0580 (5)	0.6317 (5)	0.0544 (18)	
H1A	0.8087	0.0153	0.6473	0.065*	
H1B	0.6711	0.0386	0.5791	0.065*	
C2	0.6497 (8)	0.0638 (5)	0.7001 (5)	0.058 (2)	
H2A	0.5963	0.0094	0.7017	0.069*	
H2B	0.7139	0.0714	0.7548	0.069*	
C3	0.4362 (8)	0.1404 (5)	0.7048 (5)	0.0529 (18)	
C4	0.3344 (8)	0.2171 (5)	0.6825 (6)	0.062 (2)	
H4A	0.2652	0.2132	0.7178	0.074*	
H4B	0.2838	0.2095	0.6242	0.074*	
C5	0.3984 (8)	0.3118 (5)	0.6917 (5)	0.057 (2)	
C6	0.5430 (8)	0.4094 (4)	0.6161 (5)	0.0547 (19)	
H6A	0.6065	0.4242	0.6693	0.066*	
H6B	0.4695	0.4544	0.6038	0.066*	
C7	0.6230 (8)	0.4061 (4)	0.5466 (5)	0.0537 (19)	
H7A	0.5573	0.4032	0.4920	0.064*	
H7B	0.6802	0.4593	0.5479	0.064*	
C8	0.8332 (8)	0.3265 (5)	0.5371 (4)	0.0542 (19)	
C9	0.9336 (8)	0.2487 (5)	0.5576 (6)	0.062 (2)	
H9A	0.9899	0.2582	0.6142	0.075*	
H9B	0.9981	0.2515	0.5192	0.075*	
C10	0.8769 (8)	0.1547 (5)	0.5543 (5)	0.061 (2)	
C11	0.3854 (11)	0.0643 (7)	0.7509 (7)	0.097 (4)	
H11A	0.4654	0.0305	0.7801	0.146*	
H11B	0.3242	0.0268	0.7109	0.146*	
H11C	0.3343	0.0871	0.7911	0.146*	
C12	0.2761 (10)	0.3782 (7)	0.6769 (7)	0.094 (3)	
H12A	0.2261	0.3753	0.6187	0.141*	
H12B	0.3126	0.4371	0.6894	0.141*	
H12C	0.2124	0.3639	0.7131	0.141*	
C13	0.4937 (10)	0.3250 (7)	0.7790 (5)	0.075 (3)	
H13A	0.5787	0.2907	0.7832	0.113*	
H13B	0.4448	0.3059	0.8216	0.113*	
H13C	0.5177	0.3868	0.7871	0.113*	
C14	0.8869 (11)	0.4047 (7)	0.4960 (7)	0.098 (4)	
H14A	0.8925	0.4556	0.5325	0.146*	
H14B	0.8233	0.4172	0.4429	0.146*	
H14C	0.9793	0.3915	0.4863	0.146*	
C15	1.0026 (10)	0.0905 (6)	0.5757 (7)	0.086 (3)	
H15A	0.9682	0.0305	0.5735	0.128*	
H15B	1.0565	0.1030	0.6317	0.128*	
H15C	1.0618	0.0978	0.5355	0.128*	
C16	0.7851 (10)	0.1329 (7)	0.4671 (5)	0.083 (3)	
H16A	0.7482	0.0736	0.4677	0.124*	
H16B	0.8415	0.1369	0.4251	0.124*	
H16C	0.7080	0.1744	0.4539	0.124*	
N1	0.7908 (6)	0.1469 (3)	0.6206 (3)	0.0406 (12)	
H1	0.8530	0.1580	0.6704	0.049*	
N2	0.5521 (6)	0.1404 (3)	0.6823 (3)	0.0417 (13)	
N3	0.4791 (5)	0.3212 (3)	0.6221 (3)	0.0374 (12)	
H3	0.4139	0.3140	0.5728	0.045*	
N4	0.7147 (6)	0.3268 (3)	0.5588 (3)	0.0390 (12)	
Cl1	0.2531 (2)	0.35472 (13)	0.42578 (14)	0.0632 (5)	
Cl2	0.4140 (3)	0.15858 (13)	0.47320 (12)	0.0673 (6)	
Cl3	0.5249 (2)	0.36196 (14)	0.31834 (14)	0.0662 (6)	
Cl4	0.3711 (3)	0.17021 (12)	0.25925 (12)	0.0684 (6)	
Cu1	0.63166 (8)	0.23288 (5)	0.61849 (5)	0.0404 (3)	
Cu2	0.39080 (9)	0.26064 (5)	0.36943 (5)	0.0452 (3)	

### Atomic displacement parameters (Å^2^)

**Table:**

	*U*^11^	*U*^22^	*U*^33^	*U*^12^	*U*^13^	*U*^23^
C1	0.065 (5)	0.033 (4)	0.063 (5)	0.009 (3)	0.011 (4)	0.007 (3)
C2	0.068 (5)	0.033 (4)	0.072 (5)	0.006 (3)	0.015 (4)	0.025 (4)
C3	0.059 (5)	0.050 (4)	0.056 (4)	−0.011 (4)	0.028 (4)	0.012 (3)
C4	0.049 (4)	0.067 (5)	0.080 (6)	−0.002 (4)	0.037 (4)	0.009 (4)
C5	0.055 (4)	0.057 (5)	0.070 (5)	0.010 (4)	0.037 (4)	−0.008 (4)
C6	0.067 (5)	0.030 (4)	0.064 (5)	0.009 (3)	0.007 (4)	0.003 (3)
C7	0.067 (5)	0.030 (4)	0.063 (5)	−0.009 (3)	0.011 (4)	0.012 (3)
C8	0.060 (5)	0.063 (5)	0.041 (4)	−0.019 (4)	0.015 (4)	0.005 (3)
C9	0.055 (5)	0.064 (5)	0.078 (6)	−0.001 (4)	0.037 (4)	0.004 (4)
C10	0.058 (5)	0.064 (5)	0.072 (5)	0.003 (4)	0.035 (4)	−0.017 (4)
C11	0.087 (7)	0.087 (7)	0.131 (9)	−0.009 (6)	0.052 (7)	0.052 (7)
C12	0.079 (7)	0.075 (7)	0.142 (10)	0.024 (5)	0.055 (7)	−0.012 (6)
C13	0.082 (6)	0.092 (7)	0.060 (5)	−0.002 (5)	0.034 (5)	−0.020 (5)
C14	0.085 (7)	0.096 (8)	0.124 (9)	−0.017 (6)	0.051 (7)	0.045 (7)
C15	0.073 (6)	0.070 (6)	0.128 (9)	0.015 (5)	0.054 (6)	−0.023 (6)
C16	0.090 (7)	0.104 (8)	0.061 (5)	−0.005 (6)	0.033 (5)	−0.020 (5)
N1	0.048 (3)	0.037 (3)	0.035 (3)	0.004 (2)	0.005 (2)	−0.004 (2)
N2	0.058 (4)	0.027 (3)	0.042 (3)	0.002 (2)	0.015 (3)	0.007 (2)
N3	0.043 (3)	0.028 (3)	0.039 (3)	0.004 (2)	0.003 (2)	−0.002 (2)
N4	0.051 (3)	0.033 (3)	0.032 (3)	−0.004 (2)	0.007 (2)	0.007 (2)
Cl1	0.0649 (12)	0.0523 (11)	0.0735 (13)	0.0108 (9)	0.0173 (10)	−0.0089 (9)
Cl2	0.1047 (17)	0.0421 (10)	0.0533 (11)	−0.0027 (10)	0.0129 (11)	0.0037 (8)
Cl3	0.0785 (14)	0.0529 (12)	0.0718 (13)	−0.0212 (10)	0.0261 (11)	−0.0083 (9)
Cl4	0.1169 (18)	0.0369 (10)	0.0491 (10)	−0.0053 (10)	0.0125 (11)	−0.0054 (8)
Cu1	0.0538 (5)	0.0234 (4)	0.0523 (5)	0.0064 (3)	0.0297 (4)	0.0089 (3)
Cu2	0.0550 (6)	0.0330 (5)	0.0470 (5)	−0.0004 (4)	0.0099 (4)	−0.0036 (3)

### Geometric parameters (Å, º)

**Table:**

C1—N1	1.477 (8)	C10—C16	1.529 (12)
C1—C2	1.499 (10)	C10—C15	1.531 (12)
C1—H1A	0.9700	C11—H11A	0.9600
C1—H1B	0.9700	C11—H11B	0.9600
C2—N2	1.478 (8)	C11—H11C	0.9600
C2—H2A	0.9700	C12—H12A	0.9600
C2—H2B	0.9700	C12—H12B	0.9600
C3—N2	1.250 (9)	C12—H12C	0.9600
C3—C11	1.504 (10)	C13—H13A	0.9600
C3—C4	1.508 (11)	C13—H13B	0.9600
C4—C5	1.547 (11)	C13—H13C	0.9600
C4—H4A	0.9700	C14—H14A	0.9600
C4—H4B	0.9700	C14—H14B	0.9600
C5—N3	1.507 (8)	C14—H14C	0.9600
C5—C13	1.521 (12)	C15—H15A	0.9600
C5—C12	1.526 (11)	C15—H15B	0.9600
C6—N3	1.473 (8)	C15—H15C	0.9600
C6—C7	1.495 (10)	C16—H16A	0.9600
C6—H6A	0.9700	C16—H16B	0.9600
C6—H6B	0.9700	C16—H16C	0.9600
C7—N4	1.474 (9)	N1—Cu1	2.003 (5)
C7—H7A	0.9700	N1—H1	0.9100
C7—H7B	0.9700	N2—Cu1	1.982 (5)
C8—N4	1.268 (9)	N3—Cu1	1.994 (5)
C8—C14	1.497 (10)	N3—H3	0.9100
C8—C9	1.510 (11)	N4—Cu1	1.975 (5)
C9—C10	1.513 (11)	Cl1—Cu2	2.263 (2)
C9—H9A	0.9700	Cl2—Cu2	2.249 (2)
C9—H9B	0.9700	Cl3—Cu2	2.267 (2)
C10—N1	1.496 (9)	Cl4—Cu2	2.216 (2)
			
N1—C1—C2	108.4 (6)	C5—C12—H12A	109.5
N1—C1—H1A	110.0	C5—C12—H12B	109.5
C2—C1—H1A	110.0	H12A—C12—H12B	109.5
N1—C1—H1B	110.0	C5—C12—H12C	109.5
C2—C1—H1B	110.0	H12A—C12—H12C	109.5
H1A—C1—H1B	108.4	H12B—C12—H12C	109.5
N2—C2—C1	108.7 (5)	C5—C13—H13A	109.5
N2—C2—H2A	110.0	C5—C13—H13B	109.5
C1—C2—H2A	110.0	H13A—C13—H13B	109.5
N2—C2—H2B	110.0	C5—C13—H13C	109.5
C1—C2—H2B	110.0	H13A—C13—H13C	109.5
H2A—C2—H2B	108.3	H13B—C13—H13C	109.5
N2—C3—C11	123.5 (7)	C8—C14—H14A	109.5
N2—C3—C4	120.6 (6)	C8—C14—H14B	109.5
C11—C3—C4	115.8 (7)	H14A—C14—H14B	109.5
C3—C4—C5	117.1 (6)	C8—C14—H14C	109.5
C3—C4—H4A	108.0	H14A—C14—H14C	109.5
C5—C4—H4A	108.0	H14B—C14—H14C	109.5
C3—C4—H4B	108.0	C10—C15—H15A	109.5
C5—C4—H4B	108.0	C10—C15—H15B	109.5
H4A—C4—H4B	107.3	H15A—C15—H15B	109.5
N3—C5—C13	111.9 (6)	C10—C15—H15C	109.5
N3—C5—C12	109.1 (7)	H15A—C15—H15C	109.5
C13—C5—C12	110.7 (7)	H15B—C15—H15C	109.5
N3—C5—C4	105.9 (6)	C10—C16—H16A	109.5
C13—C5—C4	111.1 (7)	C10—C16—H16B	109.5
C12—C5—C4	107.9 (7)	H16A—C16—H16B	109.5
N3—C6—C7	108.2 (5)	C10—C16—H16C	109.5
N3—C6—H6A	110.1	H16A—C16—H16C	109.5
C7—C6—H6A	110.1	H16B—C16—H16C	109.5
N3—C6—H6B	110.1	C1—N1—C10	116.3 (5)
C7—C6—H6B	110.1	C1—N1—Cu1	105.9 (4)
H6A—C6—H6B	108.4	C10—N1—Cu1	118.7 (4)
N4—C7—C6	108.6 (5)	C1—N1—H1	104.8
N4—C7—H7A	110.0	C10—N1—H1	104.8
C6—C7—H7A	110.0	Cu1—N1—H1	104.8
N4—C7—H7B	110.0	C3—N2—C2	121.1 (6)
C6—C7—H7B	110.0	C3—N2—Cu1	128.6 (5)
H7A—C7—H7B	108.4	C2—N2—Cu1	110.3 (4)
N4—C8—C14	122.7 (8)	C6—N3—C5	115.1 (5)
N4—C8—C9	121.2 (6)	C6—N3—Cu1	106.0 (4)
C14—C8—C9	116.0 (7)	C5—N3—Cu1	117.6 (4)
C8—C9—C10	120.4 (7)	C6—N3—H3	105.7
C8—C9—H9A	107.2	C5—N3—H3	105.7
C10—C9—H9A	107.2	Cu1—N3—H3	105.7
C8—C9—H9B	107.2	C8—N4—C7	121.2 (6)
C10—C9—H9B	107.2	C8—N4—Cu1	128.2 (5)
H9A—C9—H9B	106.9	C7—N4—Cu1	110.4 (4)
N1—C10—C9	107.5 (6)	N4—Cu1—N2	178.0 (2)
N1—C10—C16	110.0 (6)	N4—Cu1—N3	85.3 (2)
C9—C10—C16	111.5 (8)	N2—Cu1—N3	94.6 (2)
N1—C10—C15	108.9 (7)	N4—Cu1—N1	94.5 (2)
C9—C10—C15	108.5 (7)	N2—Cu1—N1	85.4 (2)
C16—C10—C15	110.4 (7)	N3—Cu1—N1	177.0 (2)
C3—C11—H11A	109.5	Cl4—Cu2—Cl2	99.08 (8)
C3—C11—H11B	109.5	Cl4—Cu2—Cl1	138.65 (9)
H11A—C11—H11B	109.5	Cl2—Cu2—Cl1	95.72 (9)
C3—C11—H11C	109.5	Cl4—Cu2—Cl3	94.41 (8)
H11A—C11—H11C	109.5	Cl2—Cu2—Cl3	139.22 (10)
H11B—C11—H11C	109.5	Cl1—Cu2—Cl3	99.06 (9)
			
N1—C1—C2—N2	49.3 (8)	C13—C5—N3—C6	−62.1 (8)
N2—C3—C4—C5	−42.2 (11)	C12—C5—N3—C6	60.8 (9)
C11—C3—C4—C5	140.7 (8)	C4—C5—N3—C6	176.7 (6)
C3—C4—C5—N3	70.3 (9)	C13—C5—N3—Cu1	63.9 (7)
C3—C4—C5—C13	−51.4 (9)	C12—C5—N3—Cu1	−173.2 (6)
C3—C4—C5—C12	−172.9 (7)	C4—C5—N3—Cu1	−57.2 (7)
N3—C6—C7—N4	−49.2 (8)	C14—C8—N4—C7	−1.5 (11)
N4—C8—C9—C10	36.9 (12)	C9—C8—N4—C7	173.8 (7)
C14—C8—C9—C10	−147.5 (8)	C14—C8—N4—Cu1	−176.6 (7)
C8—C9—C10—N1	−63.7 (10)	C9—C8—N4—Cu1	−1.3 (10)
C8—C9—C10—C16	56.8 (10)	C6—C7—N4—C8	−147.9 (6)
C8—C9—C10—C15	178.6 (7)	C6—C7—N4—Cu1	27.9 (7)
C2—C1—N1—C10	−179.8 (6)	C8—N4—Cu1—N3	173.5 (6)
C2—C1—N1—Cu1	−45.5 (6)	C7—N4—Cu1—N3	−1.9 (4)
C9—C10—N1—C1	−176.4 (6)	C8—N4—Cu1—N1	−3.4 (6)
C16—C10—N1—C1	62.1 (9)	C7—N4—Cu1—N1	−178.9 (4)
C15—C10—N1—C1	−59.0 (8)	C3—N2—Cu1—N3	7.4 (7)
C9—C10—N1—Cu1	55.3 (8)	C2—N2—Cu1—N3	−174.3 (5)
C16—C10—N1—Cu1	−66.2 (8)	C3—N2—Cu1—N1	−175.6 (7)
C15—C10—N1—Cu1	172.7 (5)	C2—N2—Cu1—N1	2.6 (5)
C11—C3—N2—C2	−0.1 (12)	C6—N3—Cu1—N4	−24.4 (4)
C4—C3—N2—C2	−177.0 (7)	C5—N3—Cu1—N4	−154.8 (5)
C11—C3—N2—Cu1	177.9 (7)	C6—N3—Cu1—N2	153.5 (4)
C4—C3—N2—Cu1	1.1 (11)	C5—N3—Cu1—N2	23.1 (5)
C1—C2—N2—C3	149.9 (7)	C1—N1—Cu1—N4	−158.3 (4)
C1—C2—N2—Cu1	−28.5 (7)	C10—N1—Cu1—N4	−25.3 (5)
C7—C6—N3—C5	177.8 (6)	C1—N1—Cu1—N2	23.8 (4)
C7—C6—N3—Cu1	46.0 (6)	C10—N1—Cu1—N2	156.7 (5)

### Hydrogen-bond geometry (Å, º)

**Table:**

*D*—H···*A*	*D*—H	H···*A*	*D*···*A*	*D*—H···*A*
N1—H1···Cl3^i^	0.91	2.62	3.498 (6)	163
N1—H1···Cl4^i^	0.91	2.94	3.527 (5)	123
N3—H3···Cl1	0.91	2.62	3.479 (6)	159
N3—H3···Cl2	0.91	2.84	3.394 (5)	121

Symmetry code: (i) *x*+1/2, −*y*+1/2, *z*+1/2.
